# Whole Exome Sequencing and Molecular Modeling of a Missense Variant in* TNFAIP3* That Segregates with Disease in a Family with Chronic Urticaria and Angioedema

**DOI:** 10.1155/2018/6968395

**Published:** 2018-02-22

**Authors:** Antoneicka L. Harris, Patrick R. Blackburn, John E. Richter, Jennifer M. Gass, Thomas R. Caulfield, Ahmed N. Mohammad, Paldeep S. Atwal

**Affiliations:** ^1^Department of Cancer Biology, Mayo Clinic, Jacksonville, FL, USA; ^2^Center for Individualized Medicine, Mayo Clinic, Jacksonville, FL, USA; ^3^Department of Health Sciences Research, Mayo Clinic, Jacksonville, FL, USA; ^4^Department of Clinical Genomics, Mayo Clinic, Jacksonville, FL, USA; ^5^Department of Neuroscience, Mayo Clinic, Jacksonville, FL, USA

## Abstract

Chronic urticaria is a common condition characterized by recurrent hives lasting several weeks or months and is usually idiopathic. Approximately half of the individuals with chronic urticaria will present with episodes of angioedema that can be severe and debilitating. In this report, we describe a 47-year-old Hispanic male who presented initially for an evaluation of chronic hives following hospitalization due to hive-induced anaphylaxis. The individual had a history significant for urticaria and angioedema beginning in his early 30s. Interestingly, both the individual's 41-year-old sister and 12-year-old daughter were also affected with chronic urticaria and severe angioedema. Whole exome sequencing of the proband and several family members revealed a heterozygous variant of uncertain significance in exon 2 of* TNFAIP3*, denoted as c.65G>A (p.R22Q), in all affected members. Variants in* TNFAIP3* have been associated with multiple autoimmune diseases, susceptibility to allergy and asthma, and periodic fever syndromes, suggesting that this variant could potentially play a role in disease.

## 1. Introduction

Chronic urticaria is a common skin disorder characterized by recurrent hives lasting more than six weeks or hives that recur over several months or years [1, 2]. It is mainly a disease of adults with women being more affected than men [3]. Chronic urticaria is usually idiopathic, arising spontaneously without a stimulus. Other common types include physical urticarias that are caused by physical stimuli such as cold, heat, or pressure. Various types of urticaria may coexist together, which is not uncommon [3]. Angioedema is a swelling of the deeper epidermal and dermal layers of the skin. The association between chronic urticaria and angioedema is well established [4, 5]. Genetic factors may play a role in chronic idiopathic urticaria, but the mechanism of disease is largely unknown [6]. In this report, we describe a patient with a several-year history of chronic hives, angioedema, skin flushing, and swelling. Whole exome sequencing (WES) did not detect any causative variants in known disease genes but did identify a variant in tumor necrosis factor-alpha-induced protein 3 (*TNFAIP3*, known commonly as A20) that is possibly associated with the reported phenotype.

## 2. Case History

A 47-year-old Hispanic male was initially referred to the Mayo Clinic at 34 years of age due to severe episodes of angioedema (occurring every three to four months) and history of chronic urticaria. The initial onset of urticaria occurred in 2002 while the proband was living in Italy. He has since relocated to the United States, where he sought treatment for his urticaria flares. The proband reported that episodes of urticaria occur when his skin is exposed to hot or cold temperatures or significant pressure.

The proband tried numerous treatment modalities to suppress his urticaria, including the following antihistamines: levocetirizine, cetirizine, fexofenadine, and loratadine. Prednisone and steroid injections were the only treatments that cleared the patient's hives; however, treatment with prednisone was eventually stopped due to side effects. His elevated levels of IgE suggested that the hives were autoimmune in nature, though the accompanying tryptase elevation led to the consideration of mastocytosis. The patient had a normal bone marrow biopsy (differential and aspirate), a complete blood count (CBC) with differential within normal limits, and a normal mast cell panel (CD2, CD25, CD69, and CD117). His anti-IgE receptor antibody was elevated at 18.5% (reference range: <13%) and his C1q complement was elevated at 43 (12–22 mg/dL). He also had a normal TSH of 2.0 (>20 years: 0.3–4.2 mIU/L) and serum protein electrophoresis that was within normal limits. His tryptase levels were consistently elevated and ranged from 13.9 and 19.2 (<11.5 ng/mL), indicating mast cell activation. Due to the absence of mast cells in the bone marrow biopsy, the patient did not meet diagnostic criteria for mastocytosis and was ultimately diagnosed with chronic urticaria. At 37 years of age, the patient began experiencing “attacks” approximately every two weeks, which he described as involving hives, fever, weakness, diaphoresis, and diarrhea. The patient also experienced episodes of dizziness when standing after walking only a few feet.

**Figure 1:**
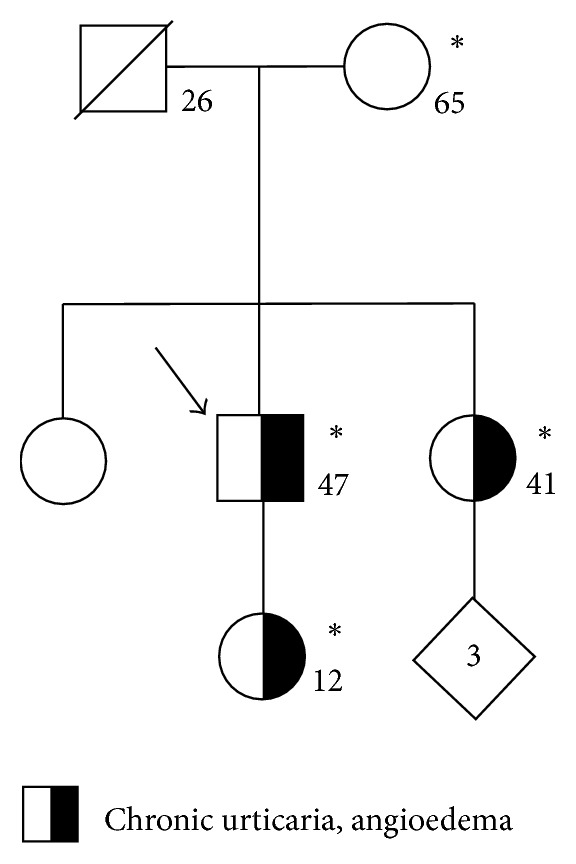
Family pedigree. Standard pedigree symbols are used. An arrow indicates the proband. The numbers inside the symbols represent the number of family members. The numbers at the right lower side of the symbols represent current age or age at death of the individuals. Asterisks indicate individuals that underwent whole exome sequencing.

Upon ascertainment of family history, we found that one of the proband's sisters and his daughter both shared his symptoms of hives and angioedema (Figure 1). The proband's daughter, who was 12 years of age at the time of evaluation, had episodes of hives and swelling of her hands and feet. She participated in gymnastics but had to discontinue this activity due to swelling of her feet after practice. She has also developed hand swelling after playing the cello.

## 3. Methods

### 3.1. Whole Exome Sequencing

Using genomic DNA extracted from the proband, his mother, sister, and daughter, the Agilent Clinical Research Exome kit was used to target exonic regions and flanking splice junctions of the genome. These regions were sequenced by massively parallel sequencing on an Illumina HiSeq with 100 bp paired-end reads (mean depth of coverage: 119x; quality threshold: 94.9%). Bidirectional sequence was assembled, aligned to reference gene sequences based on human genome build GRCh37/UCSC hg19, and analyzed for sequence variants using a custom-developed analysis tool (Xome Analyzer, GeneDx). Capillary sequencing was used to confirm the presence or absence of all potentially pathogenic variants identified in both the proband and relative samples.

### 3.2. Structural Modeling

TNFAIP3, also known as A20, is an ubiquitin-editing enzyme, which has dual roles in ubiquitin ligase and deubiquitinase activity. The N-terminal domain of TNFAIP3 contains an ovarian tumor (OTU) domain, involved in deubiquitinating Lys-63-polyubiquitin chains in a number of substrates. The C-terminal domain of TNFAIP3 is composed of seven zinc finger domains that coordinate ubiquitin ligase activity, including the addition of lysine-48-linked ubiquitin chains that are necessary for targeting substrates for proteasomal degradation [7, 8].

Human TNFAIP3 sequence was taken from the NCBI Reference Accession number NG_032761 version NG_032761.1 and was used for computer-assisted modeling. Monte Carlo simulations were performed on the full-length wild type protein (791 amino acids) and the p.R22Q variant. The X-ray refinement for Monte Carlo was built using YASARA SSP/PSSM method [9–14]. The structure was relaxed to the YASARA/Amber force field using knowledge-based potentials within YASARA. The side chains and rotamers were adjusted with knowledge-based potentials, simulated annealing with explicit solvent, and small equilibration simulations using YASARA's refinement protocol [15]. The entire full-length structure was modeled, filling in any gaps or unresolved portions from the X-ray.

Refinement of the finalized models was completed using either Schrodinger's LC-MOD Monte Carlo-based module or NAMD2 protocols. These refinements started with YASARA generated initial refinement [9–11, 13]. The superposition and subsequent refinement of the overlapping regions yielded a complete model for TNFAIP3. The final structures were subjected to energy optimization with PR conjugate gradient with an R-dependent dielectric.

Atom consistency was checked for all 791 amino acids (13,028 atoms) of the full-length wild type model and 791 amino acids (13,028 atoms) for the p.R22Q variant, verifying correctness of chain name, dihedrals, angles, torsions, nonbonds, electrostatics, atom typing, and parameters. Each model was exported to the following formats: Maestro (MAE) and YASARA (PDB). Model manipulation was done with Maestro (Macromodel, version 9.8, Schrodinger, LLC, New York, NY, 2010) or Visual Molecular Dynamics (VMD) [16]. Analyses were restricted to the N-terminus region containing the first 350 amino acids given the length and C-term distance from the site of mutation.

Monte Carlo dynamics searching (LCMOD-MC) was completed on each model for conformational sampling, using methods previously described in the literature [17–20]. Briefly, each TNFAIP3 variant system was minimized with relaxed restraints using either Steepest Descent or Conjugate Gradient PR and then allowed to undergo the MC search criteria, as shown in the literature [17–20]. The primary purpose of MC, in this scenario, is to examine any conformational variability that may occur with different mutations in the region near the mutation and the possible effect on TNFAIP3 structure and function.

## 4. Results

### 4.1. Exome Sequencing Results

WES of the proband uncovered a novel variant in* TNFAIP3* (Chr6(GRCh38): g.137871292G>A; NM_001270507.1: c.65G>A; p.(Arg22Gln)), which could possibly correlate with the proband's disease phenotype. The p.R22Q variant is absent from ExAC (Exome Aggregation Consortium) and has only been observed in 1/30936 whole genomes sequenced in gnomAD (Genome Aggregation Database) [21]. Of note, this variant was seen in 1/838 Latino genomes with a predicted minor allele frequency (MAF) of 0.001193%, suggesting that it may be more common in Latino populations. The Arg22 residue is conserved across species down to zebrafish* (Danio rerio)*, and* in silico* prediction algorithms predict the variant to be deleterious (PredictSNP2 deleterious score: 1.0000; CADD deleterious score: 22.6000; FATHMM deleterious score: 0.9806) [22]. This variant results in a semiconservative amino acid substitution that may impact secondary protein structure, and more detailed studies including* in silico* molecular modeling and dynamics simulations were pursued. Based on the ACMG 2015 guidelines, the c.65G>A (p.R22Q) variant was classified as a variant of uncertain significance (VUS) [23]. The proband's mother was found to be negative for the p.R22Q variant (the proband's father was deceased), whereas both his daughter and sister (both affected) were heterozygous for the VUS. Another pathogenic variant was detected in* GJB2* (Chr13(GRCh38): g.20189547del; NM_004004.5: c.35del; p.Gly12Valfs*∗*2; exon 2) in the proband and his mother only, which may be associated with his mild hearing loss.

### 4.2. Structure-Function Results

**Figure 2:**
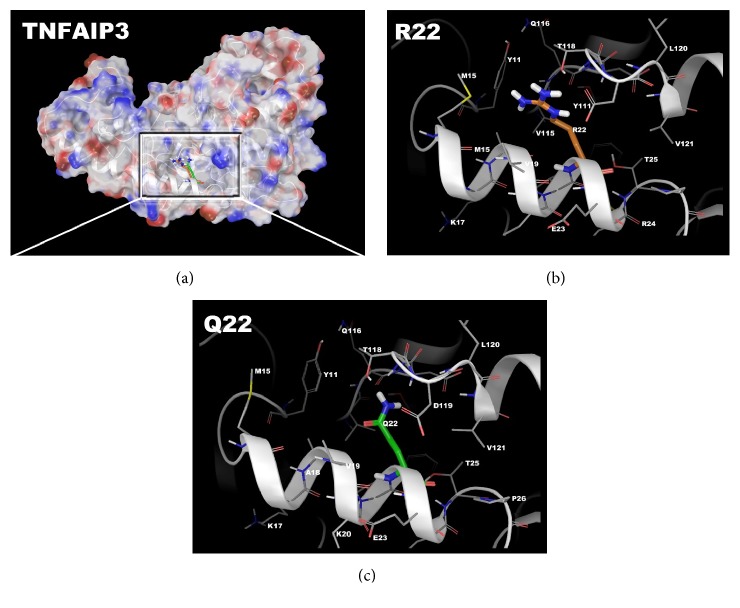
TNFAIP3 molecular model for full-length human sequence consisting of 791 amino acids and the variant p.R22Q. (a) Full-length model for the entire TNFAIP3 structure with electrostatic mapping onto the ribbon structure is shown. The density map is cut away to reveal where Arg22 and Gln22 play a role in the interaction between the helix and the loop. (b) Zoom-in on the wild type for the Arg22 residue from the full-length model to better show the interacting residues. (c) Zoom-in on the region around the Gln22 variant, showing nearby residues. All protein residues shown in licorice rendering and using standard element coloring (C-gray, O-red, N-blue, H-white, and S-yellow) except for the highlighted residues (Arg22: orange carbons, Gln22: green carbons).

When comparing the wild type and the p.R22Q variant, we found the energetic stability of the object based on Δ*G* per amino acid calculations to be comparable. The wild type object stability was 53.12 kcal/mol*∗*Å^2^, while the p.R22Q variant results in a net increase in free energy of 0.755 kcal/aa*∗*mol*∗*Å^2^, which could be locally destabilizing [17–19, 24–27]. This object stability was positive, indicating that some dynamic changes are likely with a molecular simulation for conformational sampling. Thus, we examined the local residues and determined that an electrostatic calculation may be useful to explain the change in function. The molecular models for the full structure and its variant form were generated (Figure 2) using previously described methods [17–20, 24, 27–34].

Local residues within a 12 Å cutoff near the variant site (p.R22Q) include Y11, M15, K17, V19, R22, E23, R24, T25, Y111, Q116, T118, L120, and V121, which forms around a helical region that has two interacting helices and a loop region starting at Y111 and extending to L120 (Figure 2). The R22 residue has interesting bridging interactions with the amino acids in the parallel helix, which includes Y111, T118, and V115. While the residues from the other helix are interacting with the N-terminus arginine, there is also some helix-helix interaction with T25-V121 and Y11-Q116 (Figure 2).

**Figure 3:**
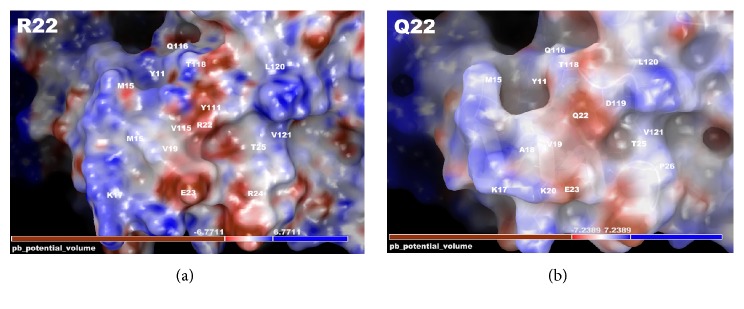
TNFAIP3 electrostatic mapping for interaction potential. Full-length model for the entire TNFAIP3 structure with electrostatics calculated using Poisson-Boltzmann (PB) calculation overlaid onto the structure. The relevant region is zoomed in to show all residues within 12 Å of residue position 22. (a) Interacting residues surrounding Arg22 are given with PB electrostatics mapped onto the protein surface. The white label gives the residues' position. The electrostatic map shows the negative to positive (red to blue) distribution and the contours and shape of the protein in this region. (b) Mutant variant p.R22Q TNFAIP3 model is given with electrostatics overlaid, indicating a muted negative charge. The depiction is similar to that in (a).

Mapping electrostatics was accomplished using the Poisson-Boltzmann calculation for solvation on the entire 791-amino-acid structure. The effects of the changes were strongly pronounced on electrostatic distribution with a +3 KT/E cutoff for both. The wild type structure (all 791 aa) shows a distinct distribution of charge around R22, which forms some definite contours for binding partner association and strengthens the domain structure. This may be useful for partner proteins or substrates (Figure 3). The p.R22Q structure is predominately negative in charge distribution at the immediate vicinity of position 22 (within 4 Å) and has a large positive distribution in a ring-like configuration around that zone (>4 Å but <12 Å) (Figure 3), which includes residues Y11, Q116, T118, L121, and K17. This kind of potential charge distribution is consistent with an electrostatic funnel that directs charge partners towards selected residues to bind [17–19, 24–27].

The electrostatics calculations from the wild type when compared with p.R22Q variant show some interesting changes due to the weakening of the Arg to Gln mutation among residues D119, T25, E23, V19, and T118, which are within interaction distance of Q22. Also of note is that distribution of the charge for the “funnel” observed (Figure 3(a)) is demonstrated to be weaker, with shallower contour surfaces and introduction of a large charge “hole” found on the far right hand side to the right of Val121 and Pro26. The positively charged ring is dissipated in the Gln22 structure, which would indicate that the effect noted above may be eliminated, potentially contributing to the altered TNFAIP3 function.

## 5. Conclusion

This report describes a 47-year-old Hispanic male with an extensive history of chronic urticaria and angioedema that various treatment modalities failed to resolve. It was initially suggested that the patient might have mastocytosis or autoimmune-related hives due to his elevated tryptase and IgE levels, respectively. Bone marrow biopsy and computerized tomography (CT) scans ruled out visceral involvement by systemic mastocytosis, and the patient was later diagnosed with chronic urticaria. The cause of his chronic urticaria is unknown; however, a history of hives in the proband's daughter and sister led to genetic testing of some of his family members. Two genes were identified through WES:* TNFAIP3* and* GJB2.*

The c.35delG variant in* GJB2 *has been reported previously in association with autosomal recessive nonsyndromic hearing loss when present in the homozygous state or when in* trans* with another pathogenic variant [35]. This variant is the most common* GJB2 *pathogenic variant among individuals of European ancestry, and the hearing loss present in individuals homozygous for the c.35delG variant ranges from mild to profound [36]. Most individuals known to be heterozygous for this variant have normal hearing, although subclinical differences in the otoacoustic emissions of carriers have been noted upon audiologic examination [37]. The proband's mother was heterozygous for the c.35delG variant in* GJB2 *while his sister and daughter did not carry this variant.

The proband, his daughter, and his sister who are all affected with chronic urticaria and angioedema each carry the c.65G>A (p.R22Q) variant in* TNFAIP3.* The variant is absent in the proband's mother who is unaffected. This variant in* TNFAIP3* has not been reported previously as pathogenic or benign to our knowledge and was classified as a VUS. However,* TNFAIP3* is known to be involved in immune and inflammatory responses signaled by cytokines, such as tumor necrosis factor-alpha and interleukin-1 beta [38]. Additionally, multiple variants within the gene have been associated with autoimmune diseases, susceptibility to allergy and asthma, and periodic fever syndromes [39].

TNFAIP3 is a ubiquitin-editing enzyme that participates in ubiquitin ligase and deubiquitinase activities. It is also known to have multiple overlapping functions between nuclear factor kappa B (NF*κ*B) and interferon regulatory factors (IRF) [8, 40]. There is evidence of cross talk between these two signaling pathways and their roles in the inflammatory and antiviral responses, which positions TNFAIP3 as a key effector of the innate immune response [41]. Recently, Zhou et al. (2016) identified individuals from six different families affected with a Behçet-like autoinflammatory syndrome (MIM: 616744) that has a variable presentation including uveitis, chorioretinal scarring, oral, gastrointestinal, and genital ulcers, gastrointestinal inflammation, polyarthritis, rash, periodic fevers, and autoinflammation with the production of autoantibodies and increased circulating proinflammatory cytokines [42]. The age of onset for this disorder is usually in the first or second decade; however, only a few cases have been reported to date.

Currently, there is no known association between missense variants in* TNFAIP3* and familial chronic urticaria with angioedema. Many patients with chronic urticaria improve with anti-IgE therapy (omalizumab), suggesting that inappropriate activation of mast cells and basophils may play a role in disease pathogenesis. It has also been proposed that some individuals with chronic urticaria may produce IgE directed against self-antigens, but evidence for this has been lacking. The proband in this study had elevated tryptase and IgE levels, suggesting that he may benefit from a trial of this therapy in the future. Interestingly, TNFAIP3 has been shown to regulate inflammation downstream of the mast cell antigen receptor module as well as the alarmin receptor, IL-33R [43]. Heger et al. (2014) showed that mast cell-specific ablation of* Tnfaip3* in mice exacerbated disease in mouse models for rheumatoid arthritis and asthma, suggesting that dysregulation of mast cell inflammatory responses via Tnfaip3 loss can contribute to disease pathology [43].

In summary, we report a novel variant in* TNFAIP3 *in three family members, which potentially correlates with a phenotype of chronic urticaria. Additional studies, including functional analyses, will be necessary to determine the role of* TNFAIP3 *variation in susceptibility to chronic urticaria.

## Additional Points

*Limitations.* Although there were no other disease-associated variants identified by WES, it is possible that this individual may harbor additional sequence variant(s) that are undetectable by this test. Chronic urticaria is also a relatively common disorder and may have a nongenetic etiology. However, cases with a strong family history may be worthy of additional study in order to identify underlying genetic mechanisms that may be dysregulated in this condition.
